# Exploration of the Conformational Scenario for α-, β-, and γ-Cyclodextrins in Dry and Wet Conditions, from Monomers to Crystal Structures: A Quantum-Mechanical Study

**DOI:** 10.3390/ijms242316826

**Published:** 2023-11-27

**Authors:** Stefano Pantaleone, Cecilia Irene Gho, Riccardo Ferrero, Valentina Brunella, Marta Corno

**Affiliations:** Dipartimento di Chimica and Nanostructured Interfaces and Surfaces (NIS) Centre, Università degli Studi di Torino, Via P. Giuria 7, 10125 Torino, Italy; stefano.pantaleone@unito.it (S.P.); cecilia.gho@polito.it (C.I.G.); riccardo.ferrero@unito.it (R.F.); valentina.brunella@unito.it (V.B.)

**Keywords:** cyclodextrin, density functional theory, conformational exploration, IR spectra

## Abstract

Cyclodextrins (CDs) constitute a class of cyclic oligosaccharides that are well recognized and largely applied in the drug delivery field, thanks to their biocompatibility, low cost, and the possibility to be derivatized in order to tune and optimize the complexation/release of the specific drug. The conformational flexibility of these systems is one of their key properties and requires a cost-effective methodology to be studied by combining the accuracy of results with the possibility of exploring a large set of conformations. In the present paper, we have explored the conformational potential energy surface of the monomers and dimers of α-, β-, and γ-cyclodextrins (i.e., 6, 7, and 8 monomeric units, respectively) by means of fast but accurate semiempirical methods, which are then refined by state-of-the-art DFT functionals. Moreover, the crystal structure is considered for a more suitable comparison with the IR spectrum experimentally recorded. Calculations are carried out in the gas phase and in water environments, applying both implicit and explicit treatments. We show that the conformation of the studied molecules changes from the gas phase to the water, even if treated implicitly, thus modifying their complexation capability.

## 1. Introduction

Cyclodextrins (CDs) are cyclic oligosaccharides consisting of α-D-glucopyranose monomeric units connected by α-(1-4) glycosidic bonds. CDs are labeled with a Greek letter according to the number of glucopyranose units forming the molecule, i.e., 6, 7, and 8, corresponding to α-, β-, and γ-cyclodextrins, respectively [1,2,3], even if much larger structures have been synthesized, with more than 30 glucopyranose units [4]. Characterized by a non-symmetric toroidal structure, wider on one side and narrower on the other, cyclodextrins exhibit a truncated cone shape. This peculiar structural feature renders CDs particularly suitable as drug delivery systems [5] and, in their specific use as such, the different number of monomeric units affects the size of the cavity, thus impacting the size of the molecule that can be hosted [3,6]. The outermost part of the CD is more hydrophilic, while the inner cavity is more lipophilic. Thanks to these major structural features, cyclodextrins are widely utilized for drug delivery applications, due to their ability to form inclusion or encapsulation complexes with a broad range of heterogenic guest molecules. Moreover, CDs are biocompatible and biodegradable, with a relatively low-cost production [7,8].

In recent years, CDs have received much attention from the scientific community in a wider variety of applicative fields: in cosmetics [9,10,11] and in the food industry for odor and taste control [12,13,14,15,16]; in the environmental sector for heavy metal and pollutant removal [17,18,19,20,21,22]; and in the chemical/pharmaceutical sector as Drug Delivery Systems (DDS), i.e., high-performance carrier materials to deliver an established amount of drug to the targeted site within a specific period of time [6,23,24,25,26,27,28]. In the last decade, many computational works have been conducted aiming at an atomistic interpretation of the inclusion and release processes for several different guest molecules [29,30,31,32,33,34,35,36], such as amino acids [37,38,39], vitamins [40], unsaturated acids [41], and antioxidants molecules [42]. The broad applicability of the CD molecule is due to its very versatile properties: it can form 1:1, 2:1, and 2:2 type complexes (even if other ratios are also possible [43]), adapting itself to the size and properties of the guest molecule, by either complexing the guest dimers of small molecules or forming host dimers with large guest molecules [44]. Moreover, hydroxyl groups can be easily functionalized in order to further improve its complexation capabilities; as an example, the derivatization made by the methyl and hydroxypropyl groups improves cyclodextrin solubility [45]. Another relevant feature of CDs is that small molecules with symmetric acidic moieties, such as carbonic, succinic, and citric acid, can be used as cross-linkers, merging the cyclodextrins to one another, to form porous structures like nanosponges and nanogels [46,47].

The high mobility of cyclodextrins seems to be one of the main characteristics contributing to the inclusion and release of the guest molecule. Specifically, all the hydroxyl and hydroxymethyl groups are free to rotate, as well as the monomeric units, which can act as a sort of flip-flop mechanism, thus opening and closing the cavity, depending on the environmental conditions of the temperature and solvent [46,47,48,49,50]. At present, systematic works exploring the potential energy surface of the cyclodextrin bare system, its dimerization reaction, and its interaction with a solvent have been mostly carried out at a molecular mechanic level by means of large molecular dynamics simulations, therefore exploring all the possible conformations of such flexible systems, without, however, reaching the accuracy of quantum mechanically based methods [39,48,50,51,52]. On the other side, Density Functional Theory (DFT) studies are limited to arbitrarily selected conformations, in many cases neglecting the solvent, or at best using implicit (i.e., structureless) models [37,38,40,41,42,49,53,54,55,56,57,58]. However, a workflow combining these two techniques is still missing in the literature. The aim of this study is to provide accurate calculations that can discriminate among similar conformers but, at the same time, to explore the complex potential energy surface of these flexible systems by considering all the possible conformers.

In the present work, we propose a systematic study of α-, β-, and γ-cyclodextrin conformations, by means of a semiempirical methodology, to explore their potential energy surfaces, thereby refining geometries at the DFT level, carefully benchmarked against several double-hybrid DFT functionals (i.e., DFT with post-HF MP2 correction).

## 2. Results and Discussion

### 2.1. Nomenclature and Workflow

In the following, the computational characterization of the three cyclodextrins, from β, to α, to γ, is presented considering the monomeric, dimeric, and crystalline structures. The monomeric units of the α-, β-, and γ-cyclodextrins were extracted from the corresponding crystallographic structures [59,60,61].

**Figure 1:**
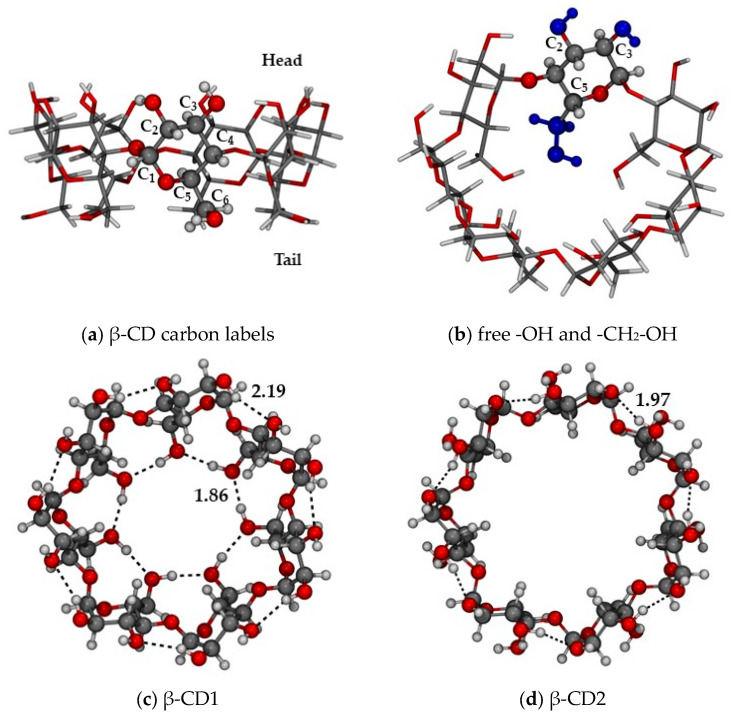
(**a**) Carbon labeling of the α-glucopyranose ring and “Head”/”Tail” parts of β-cyclodextrin; (**b**) hydroxyl and hydroxymethyl groups free to rotate during the conformational search (highlighted in blue); (**c**) closed (CD1) and (**d**) open (CD2) conformations, as optimized at the r^2^SCAN-3c level. Color code: H in white, C in grey, O in red, H-bond as red dotted lines. H-bond distances in Å.

Figure 1 reports three top and one side views of the β-cyclodextrin with the scope of describing the nomenclature adopted throughout the paper, valid also for the α and γ structures.

In Figure 1, we have highlighted the -OH-bearing carbon atoms, namely C2, C3, and C6, and we have arbitrarily labeled “Head” and “Tail” as the two parts of the molecule where the hydroxyl groups are located (C2 and C3 belong to the Head, while C6 to the Tail). Moreover, we have also reported the two possible optimized conformations, i.e., the closed and open, naming them CD1 and CD2, respectively. All the other conformations will be referred to as “xn”, where *n* corresponds to the structure number assigned during the conformational search performed by the CREST module of the xTB code (see Computational details). This nomenclature has been applied also to the cases of the α- and γ cyclodextrins, as it is defined for the monomeric glucose unit.

We sum up the computational procedure adopted for all CD monomers in the following methodological workflow:

1. *xTB-CREST* for the conformational exploration;
2. *xTB-GFN2* for semiempirical geometry optimization on all the β-CD conformations derived by CREST;
3. *Composite DFT functionals* for re-optimization on a number of structures selected in the range within 2 kcal/mol with respect to the most stable structure in the CREST energetic ranking;
4. *Double-hybrid DFT functionals* for single-point calculations during the benchmark (see Section 2.2.1);
5. *r^2^SCAN-3c composite DFT functional* for vibrational frequency calculations on a number of structures selected in the range within 1 kcal/mol with respect to the most stable structure in the r^2^SCAN-3c energetic ranking.

### 2.2. The Case of β-Cyclodextrin

#### 2.2.1. Benchmarking Computational Methods

We have chosen the β-CD molecule to perform the computational benchmark.

The conformational search was carried out on both the CD1 and CD2 conformers. The only structure obtained by CREST using the CD1 as a starting point is the CD1 itself, indicating that CREST metadynamic calculations are not able to open the fully closed conformation. All the 217 structures found by CREST during the conformational search on the CD2 were re-optimized at the GFN2 level by removing all the constraints imposed during the conformational exploration. Then, the 10 most stable structures obtained by the abovementioned conformational explorations were re-optimized with B97-3c and r^2^SCAN-3c functionals. On the two sets of obtained optimized geometries (B97-3c and r^2^SCAN-3c) single-point calculations were performed with B2PLYP-D3BJ, DSD-BLYP-D3BJ, and DSD-PBEP86-D3BJ functionals (*double-hybrid*) to crosscheck the reliability of each method from both geometrical and energetic standpoints. Indeed, the DSD functionals are reported to be very accurate in the description of isomerization reactions and non-covalent interactions [62]. In Table 1, structure labels and their relative stability values with respect to the most stable conformer (CD1) are reported. Both the B97-3c and r^2^SCAN-3c show good agreement with the double-hybrid functionals for what concerns the geometry, as the column associated with the double-hybrid methods gives good values, with a maximum error of about 1 and 3 kcal/mol, for the r^2^SCAN-3c and B97-3c, respectively. Therefore, we have selected the r^2^SCAN-3c as the functional reference for all the simulations here presented (see Table S1 for comparison with GFN2).

#### 2.2.2. β-CD Monomer in Dry and Wet Conditions

The conformational space of the β-cyclodextrin has been investigated both in the gas and aqueous phases, as this system is often used in water solvent, not only for synthesis but also during the drug delivery process in the human body. Due to the high complexity of explicitly simulating the solvent (i.e., in the presence of a great number of solvating water molecules), we have applied the C-PCM implicit model as a first approach.

**Figure 2:**
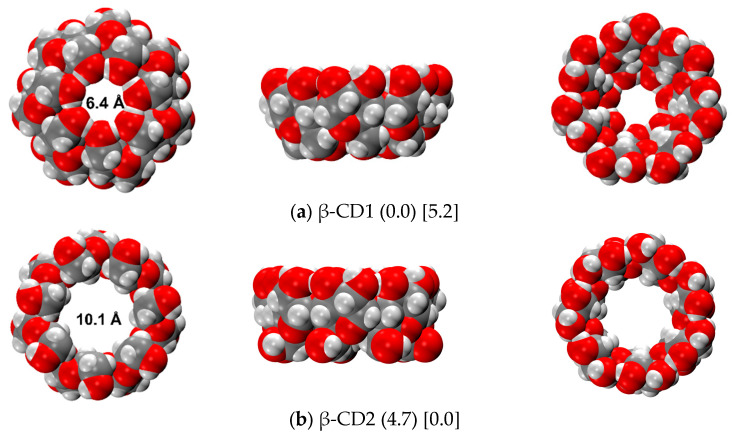
r^2^SCAN-3c optimized structures of the most stable β-CD conformers in the gas (**a**) and water (**b**) phases. Numbers in round parenthesis correspond to the relative energy in the gas phase, and in square parenthesis to the relative energy in C-PCM water (in kcal/mol). Color code: H in white, C in grey, O in red.

Figure 2 shows the two most stable structures, namely CD1 and CD2, in the gas phase and in water C-PCM. The first sports a closed configuration, in which the hydroxymethyl chains on the “Tail” side of the truncated cone are interacting with one another, forming seven strong hydrogen bonds (1.862 Å). These H-bonds are formed in addition to the H-bond pattern on the “Head” side, where all C2 hydroxyl groups donate an H-bond (2.190 Å) to the C3 of the subsequent monomeric unit (same for both CD1 and CD2). The CD1 conformation is the most stable in the gas phase, the second (CD2) being less stable at 4.7 kcal/mol (see previous section). These findings agree with a previous paper by Stachowicz et al. [49] from a qualitative point of view, even if they calculated a lower difference in energy between CD1 and CD2, equal to 2.8 kcal/mol. Moreover, in ref. [49] the authors found another CD2-type structure in which the Tail part presents seven H-bonds but with results less stable than CD1 at 18.0 kcal/mol; this specific conformation was not found by our conformational search, since CREST only retains the structures within a 6 kcal/mol range with respect to the most stable. Nevertheless, as a further check, we built that structure manually (see Figure S1) and we calculated an energy difference with respect to CD1 of 17.6 kcal/mol, very close to the literature value.

In C-PCM water solvent, the most stable structure is CD2, with all the hydroxyl moieties pointing towards the outermost part of the molecule. It is interesting to notice that the implicit solvent, despite its structureless nature, is able to deeply change the β-CD stability with respect to the gas phase. In particular, as one could expect in a real water environment, the structure changes in the sense of exposing to the solvent the most polar (hydrophilic) part of the molecule, i.e., the OH moiety of the hydroxymethyl groups, instead of the -CH_2_- aliphatic part (as in CD1). From an energetic point of view, this is the opposite with respect to the gas phase, i.e., CD2 is more stable at 5.2 kcal/mol than CD1. From a geometrical point of view, instead, the water solvation does not change the H-bond pattern of either CD1 or CD2. More details on the geometries are available in Table S2. In Table 2, the energetic ranking of the β-CD in C-PCM water is shown. For the low-lying structures within 1 kcal/mol with respect to the most stable, vibrational frequencies were also computed in order to calculate the solvation ΔG. The free energy correction does not change the relative energy ranking; however, all solvation energy values increase by ca. 10 kcal/mol because of the zero-point energy and vibrational entropy contributions that favor the open structure over the closed one. At variance with the conformational exploration in the gas phase, in the case of implicit water, the hydroxymethyl groups are much less constrained than in CD1 as they establish interactions with the solvent, thus finding many local minima.

#### 2.2.3. Increasing System Size: β-CD Dimers

To study the most stable structure of β-CD, the conformational search on the monomer is not exhaustive, as it is not certain that the dimeric form is composed of two molecules in the most stable conformation of the monomer. In this section, we report all the possible H-bonded β-CD dimers, as a full CREST analysis would be unaffordable in terms of time and memory consumption. Two possible orientations were considered: (i) the Head-Head (HH), with the two wider rings (bearing the secondary C2 and C3 hydroxyls) facing one to each other, and (ii) the opposite conformation called Tail-Tail (TT), involving the interaction between the two narrower rings, with the C6 hydroxyls (see Figure 1 for Head and Tail nomenclature). Both were built up using the most stable CD1 and CD2 conformers found for the monomers, in the gas phase and in water, respectively, for a total number of 4 structures.

**Figure 3:**
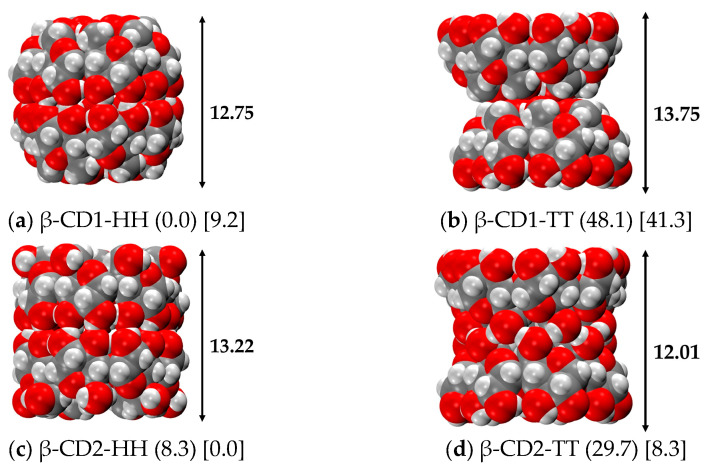
r^2^SCAN-3c optimized structures of the β-CD dimers in the gas phase. Numbers in round parentheses correspond to the relative energy in the gas phase, and in square parentheses to the relative energy in water C-PCM (in kcal/mol). H and T stand for Head and Tail sides, respectively. Color code: H in white, C in grey, O in red.

As displayed in Figure 3, the geometry of each β-CD is conserved but the dimerization has led to new structures with characteristic shapes: the CD1 Head-Head is an almost completely closed (barrel-like, Figure 3a) structure, the CD2 Head-Head is an almost perfect cylinder (Figure 3d), while both the Tail-Tail dimers have a noticeable hourglass shape (Figure 3b,c). The CD1 dimers present evident steric hindrances in the Tail side of the monomeric unit, which renders it very difficult for whatever molecule to enter within the dimer, in particular in the CD1 Tail-Tail, that presents a bottleneck in the middle of the structure (hourglass-type structure).

On the contrary, the CD2 dimers are characterized by a cavity capable of complexing several drugs, even particularly long-chain molecules, up to 13.34 Å (see Figure 3). As found for the monomer, the most stable dimer in the gas phase is represented by CD1 in the Head-Head conformation, with a dimerization energy of −64.6 kcal/mol and a dimerization free energy of −35.2 kcal/mol (see Table 3). This is the structure forming the maximum number of H-bonds, and indeed, every -OH group is engaged in two H-bonds, behaving as both donor and acceptor. On the Head side, all the hydroxyl groups on the C3 atoms (that are only H-bond acceptors in the monomer) donate an H-bond to the hydroxyl group on the C2 atom of the other β-CD, thus forming seven additional H-bonds (1.905 Å) that keep the two CDs together. The same conformation, but for CD2 (i.e., CD2-HH), is higher in energy by about 8 kcal/mol, confirming that when the two wide rings face one another, the H-bond pattern is maximized.

The TT structures are fairly less stable, about 30 to 50 kcal/mol, as they have fewer H-bond interactions; in CD1-TT, the less stable case in the gas phase and in implicit water, the two monomers are interacting only throughout dispersive forces. In a similar way as happens to the monomers, for dimers, the situation is different in the water environment and, in particular, there is an inversion of stability between the CD1 and CD2 conformations. The most stable structure in water is that of CD2-HH, not followed in the stability order by the corresponding CD1-HH, but by CD2-TT, which is stabilized with respect to the gas phase, with a dimerization energy value close to that of the most stable conformer. As expected, the less stable dimer is CD1-TT, as in the gas phase and in water its dimerization energy is even smaller (−3.5 kcal/mol). Even for dimers, the geometrical changes are, in contrast to the energies, not so affected by the implicit solvation. As a general trend, strong H-bonds (1.8–1.9 Å) tend to become slightly weaker (with an increase of 0.02–0.05 Å), while weak H-bonds (2.1–2.3 Å) become stronger (decrease of 0.1–0.3 Å).

In this case, the thermodynamic corrections deeply change the energetic features, especially if entropy is included. In the gas phase, even the relative energy is affected by entropic corrections, inverting the most stable structures, with the CD1 conformation being close and compact and, accordingly, entropically disfavored. The Gibbs free energy correction affects even more, unsurprisingly, the dimerization energy, as in that case, the entropic factor favors the reactants (2 molecules) instead of the product (1 molecule) of about 20–30 kcal/mol.

#### 2.2.4. Towards Periodicity: β-CD Crystal

**Figure 4:**
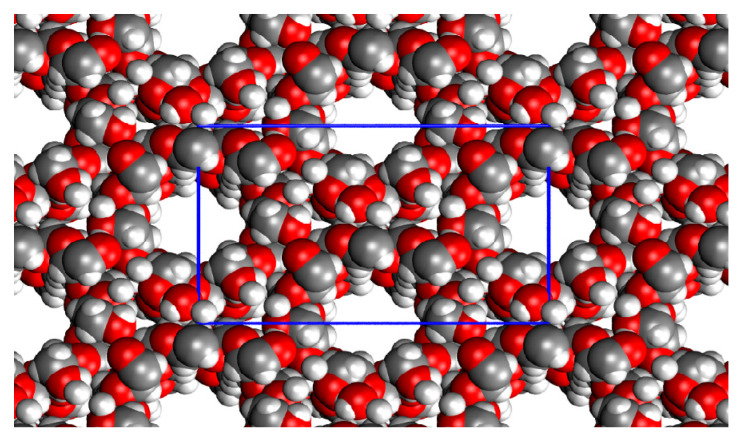
r^2^SCAN-D3BJ/def2-TZVP(gCP) optimized structure of the β-CD crystal in the gas phase (viewed in the *ab* plane). Color code: H in white, C in grey, O in red, unit cell borders in blue.

Figure 4 reports the r^2^SCAN-D3BJ/def2-TZVP(gCP) optimized crystal structure of the β-cyclodextrin, while Table 4 reports the crystallographic calculated data in comparison with the experimental ones. The spatial disposition of the cyclodextrins is made via side-by-side cylinders of the CD structures, each one composed of an alternate internal –[Head-Head-Tail-Tail]_n_- pattern and making 14 H-bonds with the neighboring CD cylinders. The percent error is high for only one cell parameter (Δ%(a) = 12.5) and, on average, the optimized structure is a little shrunk with respect to the experimental value. This is due to the fact that in the crystallographic structure, there is a very high uncertainty about the positions of the hydroxyl H atom. The first manual editing of the crystal structure was indeed devoted to redefining these specific atomic positions in a way that the H-bonds, in particular the intermolecular ones, had the maximum number possible. Due to this arbitrary decision, the final structure results are more compact than the experimental one, in which not all the hydrogens in the unit cell are in their most favorable position to maximize the number of H-bonds. The Cohesive enthalpy is larger than the dimerization energies, with the hydroxyl groups being fully engaged in H-bond interactions in all three dimensions of the periodic structure, as well as dispersive interactions. Nevertheless, the entropic corrections are similar to those of the dimers (around 30 kcal/mol).

### 2.3. α-Cyclodextrin

#### 2.3.1. α-CD Monomer

**Figure 5:**
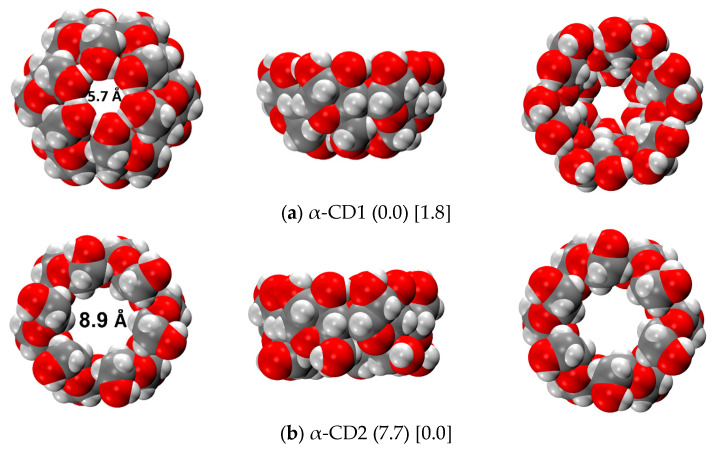
r^2^SCAN-3c optimized structures of the α-CD dimers in the gas phase. Numbers in round parentheses correspond to the relative energy in the gas phase, and in square parentheses to the relative energy in water C-PCM (in kcal/mol). Color code: H in white, C in grey, O in red.

The global minimum structure of α-cyclodextrin (α-CD) was also investigated both in the gas phase and in aqueous media, employing the same workflow as for the β-CD. In the gas phase, the CREST conformational search has only found the α-CD1 structure, as happened for β-CD. Figure 5 shows the r^2^SCAN-3c optimized structures in the gas phase, and the relative energies of α-CD1 and α-CD2 in both the gas phase and C-PCM. In this case, the α-CD2 is less stable than α-CD1 (7.7 kcal/mol) with respect to the corresponding β-CDs (4.7 kcal/mol). We attribute this difference to the major stability of the H-bonds on the Tail side of α-CD1, whose length increases from α-CD1 to β-CD1 (see Figure S2). Moreover, the shape of α-CD1 is more compact than that of β-CD1, and this increases the intramolecular dispersive interactions, further stabilizing the closed conformer over the open one. The above-mentioned stabilization is also found with the implicit solvent, as the relative energy decreases from 5.2 kcal/mol of the β-CDs to 1.8 kcal/mol of the α-CDs. At variance with the conformational exploration carried out on β-CD, which was not able to convert CD1 into CD2 and vice versa, in this case, the α-CD2 structure (used as input in the water phase) converts into CD1 during the CREST exploration procedure. Therefore, according to GFN2, the α-CD1 structure is the most stable conformer even in a water environment, as already happened for β-CD. However, r^2^SCAN-3c energies confirmed that the lowest energy conformer in the water phase is the α-CD2, as it was for β-CD. It is worth mentioning that the conformational exploration in this case returned solely α-CD2 as the only monomer contributing to the Boltzmann population, the second most stable structure being higher in energy at 4 kcal/mol (see Table S4). Probably, the reduced size of the molecule limits its mobility and, accordingly, also the stability of the various conformers, at a variance of what happens for β-CD.

#### 2.3.2. α-CD Dimer

**Figure 6:**
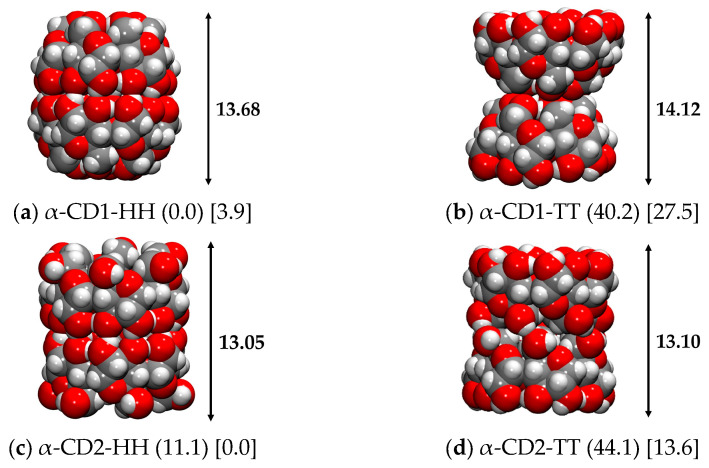
r^2^SCAN-3c optimized structures of the α-CD dimers in the gas phase. Numbers in round parenthesis correspond to the relative energy in the gas phase, and in square parenthesis to the relative energy in water C-PCM (in kcal/mol). H and T stand for Head and Tail sides. Color code: H in white, C in grey, O in red.

Following the same criteria used for the β-CD, the Head-Head and Tail-Tail dimers of α-CD were also built using the most stable CD1 and CD2 conformers found for the monomers, and their geometries were optimized in the gas phase and in implicit water. Figure 6 shows the geometrical shapes of the dimers found for α-cyclodextrin, which are very similar to those of β-CD (see Figure 3), except for the length of the α-CD1-HH dimer, which is longer than the β-CD1-HH one (13.69 Å versus 13.34 Å, respectively), as the monomer is less flattened due to the lack of one glucose unit.

The α-CD1-HH dimer has a smaller, barrel-like structure with respect to β-CD1-HH, as well as α-CD2-HH, which keeps the same cylindric structure as β-CD2-HH but with a smaller diameter. Both Tail-Tail dimers show the typical hourglass structure, which is more pronounced in the CD1 monomer. As reported in Table 5, the most stable dimer in the gas phase is represented by α-CD1-HH, with a dimerization energy of −52.0 kcal/mol and a dimerization free energy of −26.1 kcal/mol, which is a lower value compared to that of β-CD (−64.6 and −35.2 kcal/mol, see Table 3), due to the lack of one glycosidic unit and, therefore, of two H-bonds. A large difference is observed between the α- and β- Tail-Tail dimers. Specifically, the β-CD2-TT is much more stable than the β-CD1-TT (of about 20 kcal/mol) due to the presence of a strong H-bond chain at the interface between the Tail sides of the two conformers, which is completely absent in β-CD1-TT. On the contrary, the α-CD1-TT and α-CD2-TT are very close in energy (4 kcal/mol) because α-CD2-TT does not present the same strong H-bond pattern as β-CD2-TT. In implicit water, the most stable structure is that of α-CD2-HH and the dimerization energies decrease with respect to the gas phase, as well as for the β-CD dimers, due to the competitive interactions with the solvent, and in the α-CD1-HH case the free energy correction even leads to positive dimerization energy (i.e., repulsive).

### 2.4. γ-Cyclodextrin

#### 2.4.1. γ-CD Monomer

**Figure 7:**
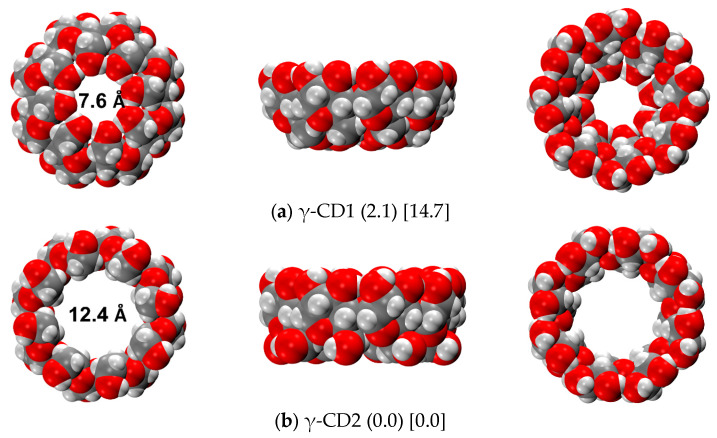
r^2^SCAN-3c optimized structures of the γ-CD dimers in the gas phase. Numbers in round parentheses correspond to the relative energy in the gas phase, and in square parentheses to the relative energy in water C-PCM (in kcal/mol). Color code: H in white, C in grey, O in red.

The most stable structure of γ-cyclodextrin (γ-CD) was also investigated both in the gas phase and in an aqueous medium, employing the same workflow previously discussed for the α and β-CDs. Figure 7 shows the well-known CD1 and CD2 conformations, very similar to the corresponding α and β-CDs, even if in this case CD2 is the most stable structure both in the gas phase and an aqueous medium, according to r^2^SCAN-3c results (while for xTB-GFN2 the most stable structure in the gas phase for all the cyclodextrins is CD1). The stability overturn of the γ-CD1 and γ-CD2 is due to the change in the H-bond pattern, particularly on the Tail side. As one can see in Figure S2, the H-bond length on the Head side decreases almost linearly from α to γ for both CD1 and CD2, while the Tail side presents an unexpected increase from β-CD1 to γ-CD1, which is indeed responsible for the minor stability of γ-CD1 with respect to the corresponding α and β-CDs.

The energetic ranking of the conformational search carried out in water is available in Table 6, as well as the solvation energies. At variance with α-CD and similar to β-CD, the conformational search in water leads to several structures with comparable energies (below 1 kcal/mol of difference), for which it is impossible to unambiguously define the most stable without performing more accurate calculations.

#### 2.4.2. γ-CD Dimer

**Figure 8:**
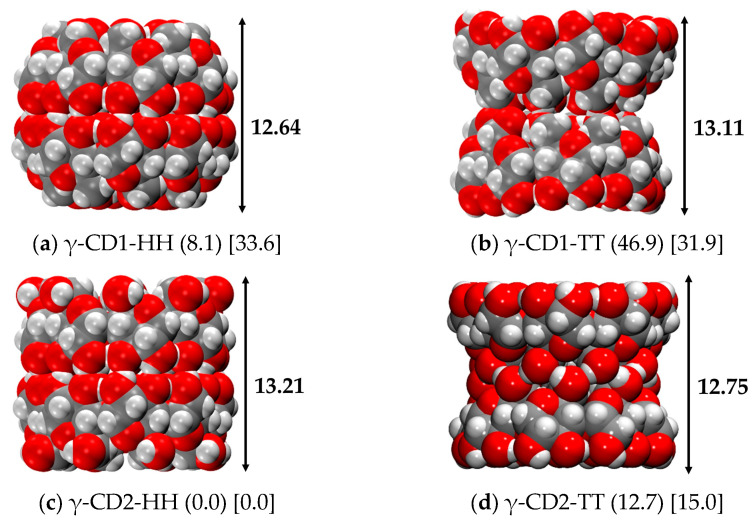
r^2^SCAN-3c optimized structures of the γ-CD dimers in the gas phase. Numbers in round parentheses correspond to the relative energy in the gas phase, and in square parentheses to the relative energy in water C-PCM (in kcal/mol). H and T stand for Head and Tail sides. Color code: H in white, C in grey, O in red.

Following the same criteria used for the α- and β-CDs, the Head-Head and Tail-Tail dimers of γ-CD were built using both CD1 and CD2 as input structures, and their stability was evaluated both in the gas and aqueous phases. Figure 8 shows the structures obtained, which are very similar to those found for the α- and β-CDs: γ-CD1-HH γ-CD2-HH keep a barrel and cylindric shape, respectively, and γ-CD1-TT and γ-CD2-TT show their typical hourglass structure. As reported in Table 7, the most stable dimer of γ-CD both in the gas and water phases is γ-CD2-HH. This is not surprising, as γ-CD2 is the most stable monomer in the gas and water phases, overcoming the structural stability exerted by the strong hydrogen bond pattern on the Head side of the γ-CD1, which is less stable at 8.1 kcal/mol. The thermodynamic corrections further penalize the CD1-HH dimer, which presents a relative Gibbs free energy of 15.5 kcal/mol, and lower the dimerization energies, as already discussed for the β- and α-CDs. In the water phase, they do not affect the relative energies, but the dimerization energies further decrease, as the CD2-TT dimer has a dimerization Gibbs free energy value of −4.8 kcal/mol (versus a dimerization energy of −37.2 kcal/mol), which is only slightly negative, showing that the dimerization process is not so favored in an aqueous medium.

### 2.5. Cyclodextrin IR-Spectra

**Figure 9:**
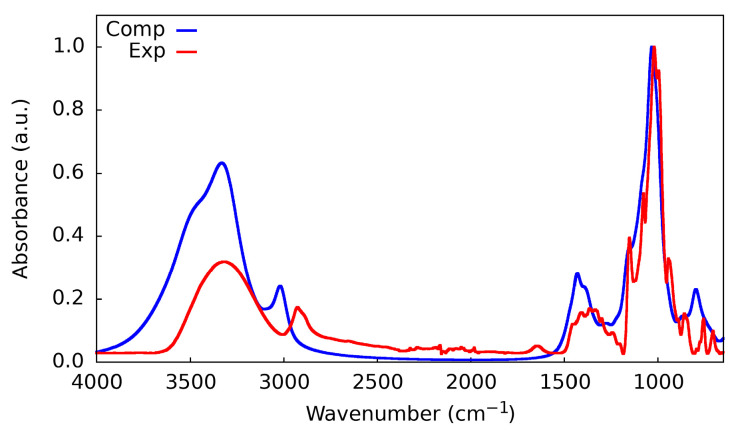
Experimental (red line) vs. computed (blue line) IR spectrum of β-CD. For the simulated spectrum, the crystal structure was used.

The experimental IR spectrum of β-cyclodextrin was recorded in order to compare it with the simulated spectrum of the crystal structure calculated at the r^2^SCAN-D3BJ/def2-TZVP(gCP) level of theory (see Figure 9 and Supporting Information for experimental details). In the Supporting Information, we have reported a comparison among the experimental spectra of α-, β-, and γ-CD, while here we discuss only that of β-CD (see Figure S3).

The experimental spectrum presents a broad band from 3000 to 3600 cm^−1^ due to the presence of many chemically different -OH bonds, as they can act as H-bond donors, H-bond acceptors, or free -OH. At approximately 3000 cm^−1^, there is the signal of the C-H bond stretching, coupled to the band from 1300 to 1450 cm^−1^ which corresponds to the bending of the same group. In the fingerprint region, it is possible to distinguish a narrow and intense peak at 1050–1100 cm^−1^, belonging to the C-C stretching. The match between simulation and experiment is good, especially in the fingerprint region, until 1500 cm^−1^; at higher wavenumbers, a red shift of the peaks is observed, mainly due to the anharmonic effects of the vibration of H-bearing moieties, which are not accounted for in the simulation. Apart from the last expected difference, the simulated IR spectrum shows that the r^2^SCAN is an accurate functional even in describing delicate properties such as vibrational frequencies and, even more, IR intensities.

### 2.6. Explicit Solvation

**Figure 10:**
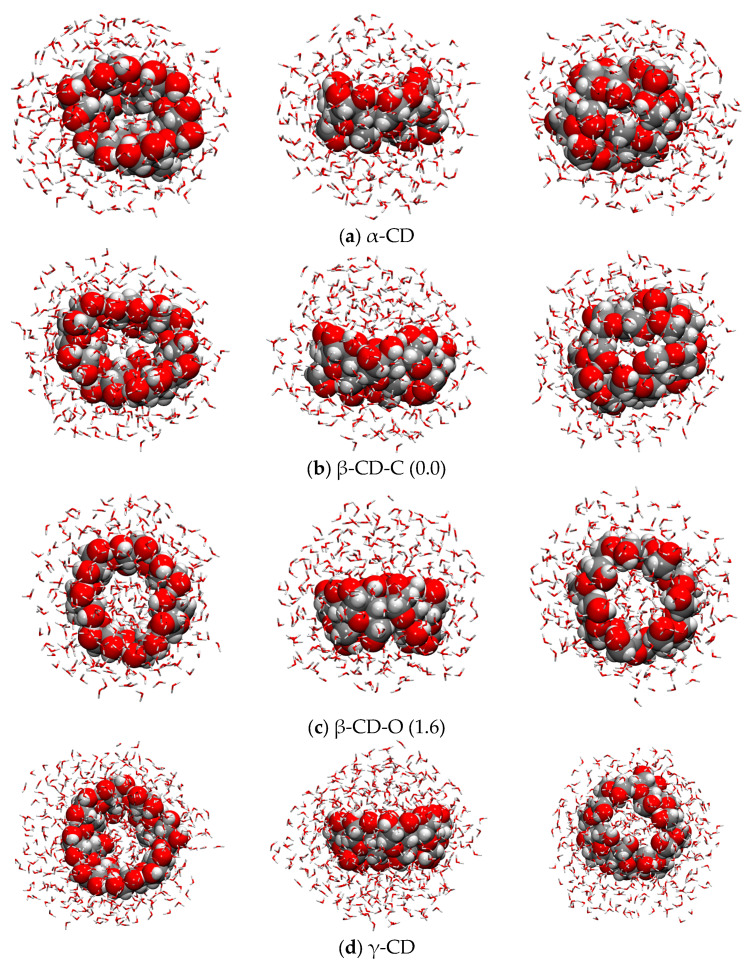
xTB-GFN2 optimized geometries of α-, β-, and γ-CDs in explicit solvation. In round parentheses are the relative energies of the two most stable β-CD conformers. In vdW representation are the cyclodextrins, and in sticks are the solvation water molecules. Color code: H in white, C in grey, O in red.

Explicit solvation was modeled throughout the QCG submodule of the CREST code. The starting structure for all the CDs was the most stable conformer in the gas phase, i.e., the closed conformation (CD1); for the water solvation shell, 300 water molecules were used for the α- and β-CDs, and this number was increased to 450 for γ-CD, in order to obtain at least a first complete solvation sphere. Not surprisingly, the explicit solvent is able to break the interactions among the -CH_2_OH groups on the Tail side, thus opening the ring (see Figure 10), which the implicit solvent cannot do. This is a further confirmation that, even if for some properties the implicit model may be enough to have an accurate description of the system, structural effects play a prominent role, particularly when the interactions between the solute and solvent are strong, as in the case of H-bonded systems.

From Figure 10, it is evident that the explicit solvation not only opens the cyclodextrins but also distorts them much more than the implicit solvent for what concerns the dihedral angles of the glycosidic units, thus losing their characteristic shape of a truncated cone. In the case of the β-CD, two stable conformers were found, in equilibrium between an open and a closed structure. The closed structure (β-CD-C, see Figure 10b) is the most stable, with the β-CD-O being 1.6 kcal/mol higher in energy. The decomposition energy performed on these systems, shown in Table S5, reveals that both the CDs and the water clusters of β-CD-O are much less stable with respect to β-CD-C ($∆E_{rel}^{CD}$ = 18.7 and $∆E_{rel}^{H_{2}O}$ = 26.9 kcal/mol); however, β-CD-O presents a stronger interaction energy ($∆E_{int}^{expl}$(β-CD-O) = −362.3 and $∆E_{int}^{expl}$(β-CD-C) = −406.3 kcal/mol) that counterbalances the lower stability of the two fragments. The small global energetic difference (1.6 kcal/mol) is due to the displacement of the enclosed water molecules which undergo unfavorable free energy with respect to the bulk water, and the value is consistent with previous calculations present in the literature (0.99 kcal/mol) [39]. This flip-flop movement of glycosidic units is believed to play a fundamental role in the complexation/release mechanisms of drugs [51].

Regarding the other cyclodextrins, for both the α- and γ-CDs, only one conformer with a non-negligible Boltzmann distribution was found (see Figure 10a,d). The α-CD (see Figure 10a) presents a completely closed conformation on the Tail side of the cavity, thus making the inclusion of a drug very difficult, and this is a possible explanation for the low usage of this specific cyclodextrin. On the contrary, γ-CD (Figure 10d) shows an open conformation on both the Head and Tail sides in which many water molecules are free to enter. It is possible that the inclusion of drug molecules is hindered in both the α- and γ-CDs because of their too-closed and open structure, respectively, while, in the case of the β-CD, the two equilibriums closed/open conformers allow for control of the inclusion/release of the drug of interest.

In Table 8, the data on implicit and explicit solvation are reported. They quantitatively converge only for the α- and β-CDs, whose implicit solvation energies are −37.9 and −49.5 kcal/mol, and explicit are −36.8 and −48.9/−41.5 (depending on the conformer), respectively. For γ-CD, the implicit solvation energy is −64.5 kcal/mol, while the explicit one is −190.5 kcal/mol, but the trend in the water affinity is kept, i.e., γ-CD > β-CD > α-CD. However, this does not correspond to the experimental evidence, i.e., that β-CD is almost insoluble (18.5 mg/L) while α- and γ-CDs dissolve much more (145 and 232 mg/L). Only in the case of normalizing the solvation energy for the solvent-accessible surface area (SASA), does the β-CD-O become less soluble than the α-CD. Probably, the relatively small complexity of these models is not enough to describe such a complex macroscopic property as the solubility, as one should also consider the possible presence of CD aggregates, thus exponentially increasing the number of atoms of the model. From Table 8, it is clear that the deformation energies can give important information: the deformation of the CDs is in a narrow energetic range (60–80 kcal/mol), while the deformation of the water cluster changes a lot depending on the CD (from 100 to 250 kcal/mol), and this is a confirmation that solvent molecules do play the most important role in determining the solubility and the conformation of a specific CD.

## 3. Materials and Methods

### 3.1. Computational Codes and Associated Parameters

#### 3.1.1. Conformational Search: xTB

Simulations with the xTB code (extended Tight-Binding) (v. 6.4.0) were carried out at the semiempirical level of theory using GFN2 [63], and at the force-field level using GFN-FF [64]. GFN2 is a generally applicable semiempirical method, which means that within a quantum mechanical scheme, some integrals are neglected and/or parametrized, thus reducing the cost of the calculation with respect to DFT by several orders of magnitude. GFN-FF, as the name suggests, is not based on quantum mechanics; in this case, only classical equations must be solved, which renders these methods much cheaper than semiempirical ones. Moreover, both GFN2 and GFN-FF include the self-consistent D4 dispersion model [65], which is useful for including dispersive interactions. The considered systems are very flexible and, in order to explore their potential energy surface, thus finding the most stable conformers, the submodule CREST (Conformer-Rotamer Ensemble Sampling Tool, v. 2.12) [66] was used at its default settings (energy convergence E_conv_ = 5 × 10^−6^ hartree, gradient convergence G_conv_ = 1 × 10^−3^ hartree·bohr^−1^, accuracy for integral cutoffs and SCF criteria = 1.0). To avoid non-physical deformations of the cyclodextrin and to save computing time, all the atoms belonging to the carbonaceous skeletons of CDs were fixed to their starting position, thus not including them in the conformational exploration. In the specific, only the moieties bound to the carbon atoms highlighted in Figure 1, i.e., the hydroxyl groups (bound to C2 and C3) and the hydroxymethyl group (bound to C5) are free to rotate. The structures obtained by CREST in the range of 6 kcal/mol (this is the default, all the structures above this threshold are not considered), i.e., those from the output file named crest_conformers.xyz, were then re-optimized at the GFN2 level using tighter thresholds to improve the accuracy of the calculations for energy convergence E_conv_ = 1 × 10^−7^ and gradient convergence G_conv_ = 2 × 10^−^⁴ (program setting: -acc 0.01). The simulations were also carried out in water, using the Analytical Linearized Poisson-Boltzmann implicit solvation model (ALPB) [67], which is parametrized for both GFN2-XTB and GFN-FF. Furthermore, the most stable cyclodextrin structures in the gas phase were treated with explicit solvation using the submodule QCG [68], which includes a docking algorithm [69] that adds water molecules, one by one, in a reasonable starting position, followed by a geometry optimization. The procedure is reiterated until the desired number of water molecules is reached. Then, the CREST program is automatically called to explore the potential energy surface of the final structure, thus finding an ensemble of the most representative low-lying energy structures, on which one can perform some statistics or refine using DFT.

#### 3.1.2. DFT Refinement: ORCA

The lowest-energy structures (within 2 kcal/mol) obtained with the xTB code (i.e., after the CREST procedure and the subsequent xTB-GFN2 optimization with tighter thresholds) were re-optimized with DFT-based methods at different levels of theory using the ORCA (v. 5.0.4) package. Two different composite DFT functionals were used: B97-3c [70] and r^2^SCAN-3c [71]. These functionals consist of a large basis set (def2-TZVP) [72] modified to optimize the efficiency of the calculations, and including some *a-posteriori* corrections: the short-range basis set incompleteness SRB (only for B97-3c), the geometrical counterpoise gCP [73] (only for r^2^SCAN-3c), and the dispersion correction (D3-BJ [74,75] and D4 [65], respectively, for B97-3c and r^2^SCAN-3c). The DFT formalism is very similar to the Hartree-Fock (HF), however, it is based on electron density, thus avoiding the large complexity of the wavefunction, thereby reaching a better accuracy than HF but at a reduced computational cost.

Three double-hybrid DFT functionals were also used to test for helping the choice between the two above-mentioned pure DFT functionals: B2PLYP [76], DSD-BLYP [77], and DSD-PBEP86 [78] (DSD stands for Dispersion corrected Spin-component Double-hybrid), coupled with a high-quality def2-QZVPP basis set [72] and D3-BJ dispersion correction [74,75]. These functionals include part of the HF formalism and the MP2 correction (one of the so-called post-HF methods), with the aim of bringing together the best features of the DFT, HF, and post-HF methodologies, but with a higher computational cost. They were chosen according to a paper by Goerigk et al. [62], which demonstrated that they were the most reliable for calculating energetic differences among conformers, thanks to an extensive benchmark on the GMTKN55 database. The double-hybrid functionals were used to test the accuracy of the cost-effective B97-3c and r^2^SCAN-3c. Since r^2^SCAN-3c showed better performances than B97-3c, we employed it for the QM calculations of all cyclodextrins we studied.

The self-consistent field (SCF) iterative procedure was converged to a tolerance in total energy of ∆E = 1 × 10^−8^ a.u. (default for geometry optimization). The thresholds on geometry optimizations were set to 1 × 10^−8^ a.u. on the energy difference from two subsequent steps, 3 × 10^−5^ and 1 × 10^−4^ hartree Bohr^−1^ on the Root Mean Square (RMS) and maximum gradients, and 6 × 10^−4^ and 1 × 10^−3^ Bohr on the RMS and maximum displacements, respectively. All the structures were also optimized in water using the C-PCM (Conductor-like Polarizable Continuum Model) implicit solvation model [79,80] (see Table S3 for comparison with different implicit solvation models). Vibrational frequencies were calculated analytically (only on the structures within a 1 kcal/mol range) to ensure that the structures are minima on the PES (i.e., all real frequencies), to include thermodynamic corrections to the electronic energy, and to simulate IR spectra.

#### 3.1.3. Periodic Simulations: CRYSTAL

Calculations on the crystal structure of β-cyclodextrin were carried out with the last public version of the CRYSTAL code, i.e., CRYSTAL23 [81,82]. Since the r^2^SCAN-3c method has not been implemented in this code yet, we have used the r^2^SCAN [83] functional coupled with a def2-TZVP basis set (which is the starting basis set modified for r^2^SCAN-3c), and with similar a-posteriori corrections with respect to the r^2^SCAN-3c, thus maintaining the same level of accuracy of molecular calculations. Specifically, we applied the gCP (geometrical counterpoise) to the selected basis set [73], and the D3-BJ (Becke-Johnson) dispersion correction [74,75]. A careful check of the r^2^SCAN-D3BJ/def2-TZVP(gCP) vs. r^2^SCAN-3c is available in the Supporting Information. For the numerical integration-correlation term of all calculations of exchange, 75 radial points and 947 angular points (XLGRID) in a Lebedev scheme in the region of chemical interest were adopted. The Pack-Monkhorst/Gilat shrinking factors for the reciprocal space were set to 2 [84]. Tolerances of the integral calculations for Coulomb overlap and Coulomb penetration were increased to 10^−7^ hartree (10^−6^ hartree is the default). The SCF iterative procedure was converged to a tolerance in total energy of ∆E = 1 × 10^−7^ hartree for geometry optimization and ∆E = 1 × 10^−11^ hartree for frequency calculations. Both the cell parameters and atomic positions were set free to relax during the geometry optimization procedure, whose thresholds on gradients and displacements were set to the default values (3 × 10^−4^ hartree bohr^−1^ and 1.2 × 10^−3^ bohr, respectively). Vibrational frequencies, referred to as the Γ point, were calculated on the optimized geometry by means of a mass-weighted Hessian matrix, which is obtained by the numerical differentiation of the analytical first derivatives [85,86]. The Berry phase approach was applied to calculate intensities in the simulation of IR spectra [87]. The full width at half maximum of the OH stretching band was chosen according to the red shift with respect to free silanols in the isolated β-CD molecule, as proposed by Pimentel and coworkers [88].

Structure editing and visualization were performed using the MOLDRAW (v. 2.0) [89] and Gaussview (v. 6) [90] programs, while figure rendering was performed using the VMD software (v. 1.9.3) [91].

#### 3.1.4. Reference Equations for Computing the Energetic Contributions

The relative energies presented in the Tables are calculated as:

$$∆E_{rel}=E_{n}-E_{0}$$

where *E_n_* is the electronic energy of the *n*^th^ conformer, and *E*_0_ is the electronic energy of the most stable conformer (that may change according to the level of theory, or if the calculation is carried out in gas or liquid phase). Similarly, the dimerization energies are calculated as:

$$∆E_{dim}=E_{dimer}-E_{0}$$

where *E_dimer_* is the electronic energy of the cyclodextrin dimer, and *E*_0_ is the electronic energy of the most stable conformer of the monomer. Solvation energies in implicit water were calculated as:

$$∆E_{solv}^{PCM}=E_{CPCM}-E_{gp}$$

where *E_CPCM_* is the absolute electronic energy in implicit solvation (vide *infra*), and *E_gp_* is the absolute energy at the same level of theory in the gas phase. The cohesive energy of the crystal structure was calculated according to the following equation:

$$∆E_{cohesiv}=\frac{E_{cry}-2*E_{GP}}{2}$$

where *E_cry_* and *E_gp_* are the absolute electronic energies of the crystal structure and the isolated molecule in the gas phase, respectively, while the normalization factor is due to the number of molecules composing the crystal structure. The solvation energies in explicit water are computed as follows:

$$∆E_{solv}^{expl}=E_{wCD}-\left(E_{CD}+E_{H_{2}O}\right)$$

where $E_{wCD}$ is the electronic energy of the complex at the r^2^SCAN-3c level, calculated on the GFN2 optimized geometry, E_CD_ is the electronic energy of the most stable structure in the gas phase, and $E_{H_{2}O}$ is the electronic energy of a cluster containing 300 (for α- and β-CD) or 450 (for γ-CD) water molecules, on which a conformational search was performed at a semiempirical level in order to find the most stable structure (vide *infra*). The deformation energies on both the CD and the water cluster were calculated as:

$$∆E_{def}^{CD}=E_{CD//wCD}-E_{CD}$$

$$∆E_{def}^{H_{2}O}=E_{H_{2}O//wCD}-E_{H_{2}O}$$

where $E_{CD//wCD}$ is the electronic energy of the CD only in the gas phase at the geometry of the complex in explicit solvation, $E_{CD}$ is the electronic energy of the most stable structure of the cyclodextrin in the gas phase (the same as in the previous equation), $E_{H_{2}O//wCD}$ is the electronic energy of the water cluster at the geometry of the complex in explicit solvation after removing the CD molecule, and $E_{H_{2}O}$ is the electronic energy of the water cluster (containing 300 and 450 water molecules).

## 4. Conclusions

In this work, we have studied the conformations of α-, β-, and γ-cyclodextrins in the gas phase and water environment by means of combined semiempirical and DFT computational approaches. Specifically, the xTB-GFN2 semiempirical method was applied in joint with an automatic tool (CREST) capable of exploring the potential energy surface of a given molecule throughout iterative cycles of metadynamics. The structures found by xTB were then refined at the r^2^SCAN-3c level, which was in turn benchmarked against several double-hybrid functionals coupled with a high-quality def2-QZVPP basis set. Moreover, we have utilized the QCG tool to automatically solvate the three cyclodextrins, followed by the CREST procedure, in order to obtain the most stable structure among the possible conformers. The dimers of the three cyclodextrins were modeled and optimized as well, to study their tendency to form aggregates and, following a rationale of increasing complexity, we considered the crystal structure with the aim of reproducing the experimental IR spectrum registered in our laboratories.

Our results show that α- and β-cyclodextrins prefer the closed conformation in the gas phase and the open one in implicit water, while the open conformation of γ-cyclodextrin is the most stable one in both cases. The dimers also follow the same tendency as that of the monomers. When water is treated explicitly, it strongly affects the CD structure, which appears much more distorted compared to the gas phase and the implicit solvation. Curiously, for the β-cyclodextrin we have found two structures very close in energy that were also discovered in previous works in the literature, which can be distinguished because of their open and closed cavity. In this specific case, one of the α-D-glucopyranose rings is responsible for opening and closing the cavity with a sort of flip-flop mechanism (see Figure 10), which seems to be the main driving force of moving the water molecules inside and outside the cavity and, as a consequence, of the complexation/release of the drug molecule. Finally, the comparison between the experimental and computed IR spectra is good, considering that the computed one does not take into account the anharmonic effects. This indicates that the r^2^SCAN is very robust both against more accurate DFT functionals and from an experimental point of view, so it can also be used for more complex systems, such as the study of the complexation/release mechanism that is currently ongoing in our laboratories.

Overall, we provide a cost-effective methodology for future works, either in our laboratories or performed by someone else on similar systems, to explore, in a fast but reliable way, the conformational complexity of flexible systems and to properly refine results with a cheap but accurate DFT method applicable to medium-sized systems.

## Figures and Tables

**Table 1:** Energetic ranking (in kcal/mol) of the structures used for the benchmark. Top part: r^2^SCAN-3c geometries; bottom part: B97-3c geometries. In parenthesis: the RMSD (Root Mean Square Deviation) with respect to the CD1 structure.

	Structures				ΔE_rel_			
	r^2^SCAN-3c	B2PLYP	DSD-BLYP	DSD-PBEP86	r^2^SCAN-3c	B2PLYP	DSD-BLYP	DSD-PBEP86
1.	CD1 (0.000)	CD1	CD1	CD1	0.0	0.0	0.0	0.0
2.	CD2 (1.110)	x121	x121	x121	4.7	6.7	5.9	6.0
3.	x121 (1.789)	x033	x026	x026	5.8	6.8	6.1	6.3
4.	x026 (1.858)	x026	x033	x033	6.3	6.8	6.1	6.3
5.	x033 (1.864)	x080	CD2	CD2	6.4	7.6	6.3	6.5
6.	x153 (1.820)	x072	x080	x080	7.3	7.6	6.8	6.8
7.	x023 (1.820)	CD2	x072	x072	7.3	7.7	6.8	6.8
8.	x043 (1.820)	x043	x043	x023	7.3	8.4	7.6	7.7
9.	x072 (2.147)	x023	x023	x043	7.4	8.4	7.6	7.7
10.	x080 (2.141)	x153	x153	x153	7.4	8.4	7.6	7.7
	**B97-3c**	**B2PLYP**	**DSD-BLYP**	**DSD-PBEP86**	**B97-3c**	**B2PLYP**	**DSD-BLYP**	**DSD-PBEP86**
1.	CD1 (0.000)	CD1	CD1	CD1	0.0	0.0	0.0	0.0
2.	x026 (1.853)	x033	x121	x121	8.6	6.7	6.0	6.2
3.	x033 (1.854)	x026	x033	x033	8.7	6.7	6.1	6.4
4.	CD2 (1.166)	x121	x026	x026	9.1	6.8	6.1	6.4
5.	x121 (1.788)	x080	CD2	CD2	9.2	7.6	6.4	6.7
6.	x023 (1.814)	x072	x080	x080	10.4	7.6	6.9	7.0
7.	x043 (1.814)	CD2	x072	x072	10.4	7.7	6.9	7.0
8.	x153 (1.822)	x043	x153	x153	10.5	8.4	7.7	7.9
9.	x072 (2.150)	x023	x023	x023	10.5	8.4	7.7	8.0
10.	x080 (2.150)	x153	x043	x043	10.6	8.4	7.7	8.0

**Table 2:** r^2^SCAN-3c energetic ranking (ΔE_rel_, in kcal/mol) of β-CD conformers in C-PCM. In parentheses are the free energies.

Structure	ΔE_rel_	Solvation E
CD2	0.0 (0.0)	−49.5 (−60.2)
x05	0.5 (0.4)	−49.0 (−59.8)
x01	0.5 (0.6)	−49.0 (−59.6)
x03	1.0 (1.2)	−48.5 (−59.1)
x02	1.6	−47.9
x08	3.1	−46.3
x06	3.2	−46.3
x23	3.6	−45.9
x16	4.3	−45.2
x15	4.3	−45.1
CD1	5.2	−44.3
x76	6.0	−44.0

**Table 3:** r^2^SCAN-3c relative (ΔE_rel_) and dimerization (ΔE_dim_) energies (in kcal/mol) of β-CD dimers in the gas phase and in water C-PCM. In parentheses are the free energies. H and T stand for Head and Tail sides.

Structure	Gas Phase		Water (C-PCM)	
	ΔE_rel_	ΔE_dim_	ΔE_rel_	ΔE_dim_
β-CD1-HH	0.0 (2.4)	−64.6 (−35.2)	9.2	−35.6
β-CD2-HH	8.3 (0.0)	−56.2 (−37.6)	0.0 (0.0)	−44.8 (−15.7)
β-CD2-TT	29.7	−34.8	8.3 (7.1)	−36.5 (−8.7)
β-CD1-TT	48.1	−16.5	41.3	−3.5

**Table 4:** Experimental vs. r^2^SCAN-D3BJ/def2-TZVP(gCP) optimized cell parameters of the β-cyclodextrin crystal structure. Cell parameters in Å, angle in degrees, and volume in Å^3^. Δ% is the percentage of error. Energies are in kcal/mol.

	*a*	*b*	*c*	β	Volume	Cohesive H	Cohesive G
r^2^SCAN-D3BJ/ def2-TZVP (gCP)	16.867	25.339	15.849	113.185	6226.575	−91.4	−64.2
EXP	19.056	24.415	15.698	109.463	6886.183	--	--
Δ%	12.5	−4.8	−2.0	−3.6	8.0	--	--

**Table 5:** r^2^SCAN-3c relative (ΔE_rel_) and dimerization (ΔE_dim_) energies (in kcal/mol) of α-CD dimers in the gas phase and in water C-PCM. In parentheses are the free energies. H and T stand for Head and Tail sides.

	Gas Phase		Water (C-PCM)	
Structure	ΔE_rel_	ΔE_dim_	ΔE_rel_	ΔE_dim_
α-CD1-HH	0.0 (0.0)	−52.0 (−26.1)	3.9 (12.3)	−34.6 (3.1)
α-CD2-HH	11.1 (3.8)	−40.9 (−22.3)	0.0 (0.0)	−38.5 (−9.2)
α-CD1-TT	40.2	−11.8	27.5	−11.0
α-CD2-TT	44.1	−7.9	13.6	−24.9

**Table 6:** r^2^SCAN-3c energetic ranking (ΔE_rel_, in kcal/mol) of γ-CD conformer in C-PCM. In parentheses are the free energies.

Structure	ΔE_rel_	Solvation E
γ-CD2	0.0 (0.0)	−64.5 (−74.7)
γ-x08	0.5 (1.9)	−64.0 (−72.8)
γ-x09	0.5 (0.5)	−63.9 (−74.2)
γ-x24	0.8 (1.3)	−63.6 (−73.4)
γ-x42	1.3 (1.0)	−63.2 (−73.6)
γ-x38	1.3 (2.2)	−63.2 (−72.4)
γ-x31	4.1	−60.4

**Table 7:** r^2^SCAN-3c relative (ΔE_rel_) and dimerization (ΔE_dim_) energies (in kcal/mol) of γ-CD dimers in the gas phase and in water C-PCM. In parentheses are the free energies. H and T stand for Head and Tail sides.

	Gas Phase		Water (C-PCM)	
Structure	ΔE_rel_	ΔE_dim_	ΔE_rel_	ΔE_dim_
γ-CD2-HH	0.0 (0.0)	−78.6 (−56.0)	0.0 (0.0)	−51.1 (−20.5)
γ-CD1-HH	8.1 (15.5)	−70.5 (−40.5)	33.6	−17.6
γ-CD2-TT	12.7	−37.2	13.9 (15.7)	−37.2 (−4.8)
γ-CD1-TT	46.9	−31.7	31.9	−19.3

**Table 8:** Solvation data of α-, β-, and γ-CDs in implicit and explicit solvation. SASA stands for solvent-accessible surface area. Implicit solvation energies refer to the most stable conformers at r^2^SCAN-3c (C-PCM) level of theory. Energies are in kcal/mol. Areas in Å^2^. Experimental solubility in mg/L.

	α-CD	β-CD-C	β-CD-O	γ-CD
$∆E_{solv}^{PCM}$	−37.91	−49.46		−64.48
SASA (PCM)	3014.21	3486.24		3994.49
Normalized $∆E_{solv}^{PCM}$	−1.26 × 10^−2^	−1.42 × 10^−2^		−1.61 × 10^−2^
$∆E_{solv}^{expl}$	−36.82	−48.91	−41.52	−190.53
SASA (explicit)	2759.61	3272.97	3438.82	3827.84
Normalized $∆E_{solv}^{expl}$	−1.33 × 10^−2^	−1.57 × 10^−2^	−1.28 × 10^−2^	−4.98 × 10^−2^
$∆E_{def}^{CD}$	78.30	63.51	83.39	70.34
$∆E_{def}^{H_{2}O}$	198.13	222.91	252.90	103.42
Experimental solubility	145	18.5	18.5	232

## Data Availability

Data is contained within the article and Supplementary Materials.

## Supplementary Materials

The following supporting information can be downloaded at: https://www.mdpi.com/article/10.3390/ijms242316826/s1**.**
