# Supplementary material for: Exploration of the Conformational Scenario for α-, β-, and γ-Cyclodextrins in Dry and Wet Conditions, from Monomers to Crystal Structures: A Quantum-Mechanical Study
Source: Int J Mol Sci. 2023 Nov 27;24(23):16826. doi: 10.3390/ijms242316826 (PMC10706634; doi:10.3390/ijms242316826)
Supplement: Supplementary file 1 [file ijms-24-16826-s001.zip › ijms-2667620-supplementary.pdf]

Supplementary Information

Exploration of the Conformational Scenario for α-, β-, and γ-Cyclodextrins in Dry and Wet Conditions, from Monomers to Crystal Structures: A Quantum-Mechanical Study

Stefano Pantaleone †, Cecilia Irene Gho †,‡, Riccardo Ferrero, Valentina Brunella and Marta Corno *

Dipartimento di Chimica and Nanostructured Interfaces and Surfaces (NIS) Centre, Università degli Studi di Torino, Via P. Giuria 7, 10125 Torino, Italy; stefano.pantaleone@unito.it (S.P.); cecilia.gho@polito.it (C.I.G.); riccardo.ferrero@unito.it (R.F.); valentina.brunella@unito.it (V.B.)

* Correspondence: marta.corno@unito.it; Tel.: +39-011-670-2439

† These authors contributed equally to this work.

‡ Current address: Dipartimento di Scienza Applicata e Tecnologia, Politecnico di Torino, C.so Duca degli Abruzzi 24, 10129 Torino, Italy.

Experimental details on the recorded IR spectra

FTIR spectra were recorded in ATR mode with a PerkinElmer (Waltham, MA, USA) Spectrum 100 with DTGS (triglycine sulfate deuterated) detector. Parameters used were: spectral window from 4000 to 650 cm^{-1}, resolution 4 cm^{-1}, pressure applied on sample 100 N and using 16 scan per spectrum. The FTIR spectra were performed on a powder sample of α-, β- and γ-cyclodextrin, donated by Roquette Italia S.p.A., Cassano Spinola (AL) and Roquette Frères, Lestrem (France). Powders were dried before use with oven treatment at 90 °C to remove any traces of water and then used without further purification.

Comparison xTB vs r^{2}SCAN-3c

Table S1: xTB-GFN2 and r^{2}SCAN-3c energetic ranking of the benchmark on β cyclodextrin. Energies are in kcal/mol.

Structures		Relative E	
xTB	r^{2}SCAN-3c	xTB	r^{2}SCAN-3c
CD1	CD1	0.0	0
x026	CD2	8.7	4.7
x033	x121	8.7	5.8
CD2	x026	10.8	6.3
x153	x033	12.1	6.4
x043	x153	12.1	7.3
x023	x023	12.1	7.3
x121	x043	12.4	7.3
x072	x072	13.4	7.4
x080	x080	13.4	7.4

r^2SCAN-D3BJ/def2-TZVP(gCP) vs r^2SCAN-3c

As the r^2SCAN-3c composite method has not been implemented yet in the CRYSTAL code, we calculated the energetic difference between β-CD1 and β-CD2 with r^2SCAN-3c and with r^2SCAN-D3BJ/def2-TZVP(gCP), which represents the most similar computational setup with respect to r^2SCAN-3c available in CRYSTAL. The results show that the two methods indeed provide a very close energy difference: $\Delta E_{r^2SCAN-3c}^{CD2-CD1} = 4.7\ kcal/mol$ and $\Delta E_{r^2SCAN-D3BJ/def2-TZVP(gCP)}^{CD2-CD1} = 4.1\ kcal/mol$.

H-bond interactions in α-, β-, γ-cyclodextrins

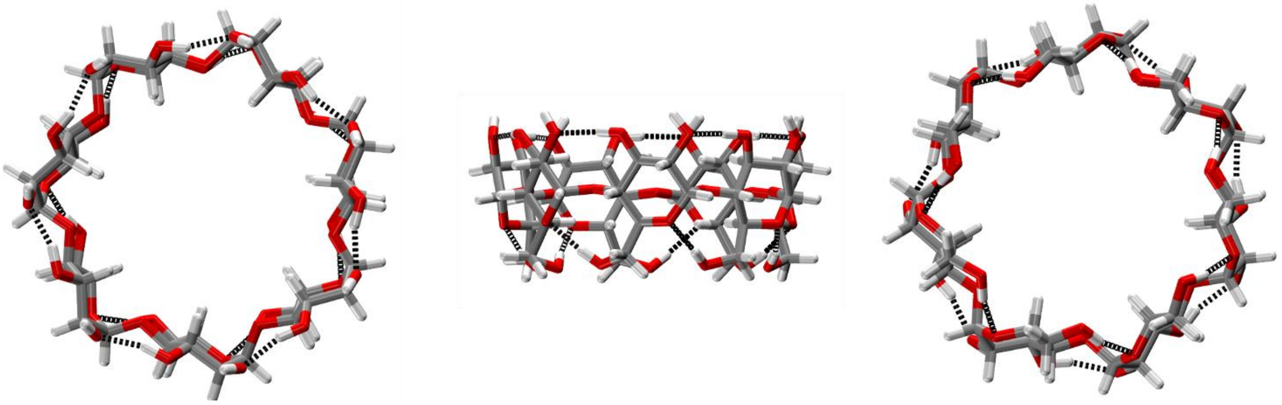

Figure S1: Top, side and bottom views of the CD2 structure of β-cyclodextrin which still presents 7 hydrogen bonds on the Tail side.

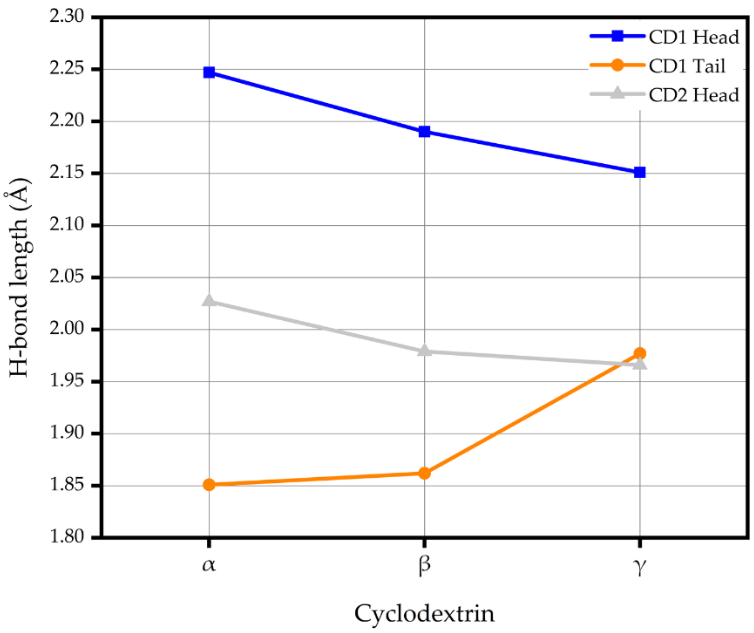

Figure S2: H-bonds length of the α- , β- and γ- cyclodextrins.

Table S2: Averaged H-bond length of cyclodextrin structures in gas phase (GP) and in implicit water (C-PCM). Distances and Root Mead Square Deviations (RSMD) are in Å.

Monomers										
	α-CD1		α-CD2	β-CD1		β-CD2	γ-CD1		γ-CD2	
	Tail	Head	Head	Tail	Head	Head	Tail	Head	Head	
GP	1.839	2.236	2.009	1.862	2.180	1.974	1.968	2.150	1.956	
C-PCM	1.859	2.190	2.027	1.868	2.046	1.986	2.017	2.093	1.964	
RMSD	0.017628		0.121950	0.059733		0.135252	0.048571		0.211632	
Dimers										
	α-CD1-HH			α-CD1-TT		α-CD2-HH		α-CD2-TT		
	Tail	Inter	Head	Tail	Inter	Inter	Head	Head	Inter	
GP	1.859	1.956	2.041	1.808	1.945	1.927	1.856	2.008	1.970	
C-PCM	1.884	1.974	2.071	1.959	1.997	1.947	1.904	2.038	-	
RMSD	0.032142			0.809568		0.095655		1.255347		
	β-CD1-HH			β-CD1-TT		β-CD2-HH		β-CD2-TT		
	Tail	Inter	Head	Tail	Head	Inter	Head	Inter	Head	
GP	1.849	1.898	2.007	1.868	2.128	1.923	1.831	1.968	2.294	
C-PCM	1.865	1.923	2.037	1.887	2.076	1.942	1.872	1.877	2.064	
RMSD	0.078053			0.493924		0.105386		0.701916		
	γ-CD1-HH			γ-CD1-TT			γ-CD2-HH		γ-CD2-TT	
	Tail	Inter	Head	Inter	Tail	Head	Inter	Head	Inter	Head
GP	1.997	1.945	1.967	1.965	1.921	1.949	1.924	1.817	1.925	1.915
C-PCM	2.095	1.939	1.967	-	1.953	1.828	1.938	1.856	1.991	1.869
RMSD	0.056541			0.806640			0.095340		0.108624	

Implicit solvation

Three different implicit solvation models were tested. The first two columns in Table 2 of the main text show the relative r^2SCAN-3c relative energies using both the two implicit solvation models implemented in ORCA software: the conductor-like continuum polarization model (C-PCM) and the solvation model based on density (SMD). C-PCM is the standard implicit solvent scheme, which treats the solute as a molecule in a cavity with polarization charges, representing the solvent. This method was compared with SMD, which is a model using the full solute electron density without computing partial atomic charges and adds a surface tension contribute at the boundary with the solvent. It requires more computational resources, but has better performances with neutral organic systems, so the accordance of these models was checked.

Table S3: Relative energies between β-CD conformers using different implicit solvation models at r^2SCAN-3c level.

CPCM		SMD		ALPB	
x00	0.00	x00	0.00	x00	0.00
x05	0.47	x01	0.21	x01	0.82
x01	0.50	x05	0.33	x03	1.29
x03	0.96	x03	0.50	x05	1.30
x02	1.62	x02	1.50	x02	2.83
x08	3.11	x06	1.56	x08	5.19
x06	3.18	x08	2.81	x15	5.25
x23	3.60	x16	3.15	x23	7.07
x16	4.29	x23	3.24	x16	7.21
x15	4.34	x15	3.31	x06	9.14
cd1	5.19	x76	5.44	cd1	15.10
x76	6.06	cd1	7.63	x76	15.48

The third column show the relative electronic energy of r^2SCAN-3c summed to the implicit solvent contribution of ALPB, a model proposed by Grimme and co-workers recently implemented in the xTB package. GFN2 calculations gave the result that the CD1 structure was still the most stable structure even in aqueous phase (see Table S1 for a better comparison between GFN2 and r^2SCAN-3c results), with an energy difference with the CD2 structure of about 5.5 kcal/mol. The re-optimization with DFT method overturned this result, showing that CD2 structure is 5 kcal/mol lower in energy than CD1, if considering the presence of a solvent. To understand if this mistake of the software is due to electronic contribution or implicit solvation model (ALPB), a cross-check between the r^2SCAN-3c optimized structures was performed, removing the solvation contribution included by ORCA and integrating the ALPB contribution calculated by xTB. The relative energies reported in Table S3 were obtained using these new absolute energies computed as follows:

$$E_{r^2SCAN3c-ALPB} = E_{r^2SCAN3c-CPCM} - CPCM_{correction} + ALPB_{correction}$$

The order of the stability of the structures is in good agreement with the other solvation methods, although the ALPB contribution is higher. This cross-check confirmed that GFN2 method does not provide a good description of electronic energy, maybe because it overestimates the hydrogen bond contribution.

Thanks to this test, CPCM was chosen as the preferred implicit solvent method for the future aqueous phase calculation because it shows good agreement with the more recent and accurate SMD method, with lower computational demand.

Table S4: R^2SCAN-3c energetic ranking (in kcal/mol) of α-CD conformer in C-PCM.

Structure	ΔE_{rel}	Solvation E
α-CD2	0.0 (0.0)	-37.9 (-48.6)
α-CD1	1.8	-36.1
x055	4.0	-33.9

Explicit solvation

Table S5: Decomposition of the binding energies calculated as single point at R^2SCAN-3c level on the GFN2 β-CD-C and β-CD-O optimized geometries. ΔE_{rel}^{tot} is the total energetic difference between the two conformers, while ΔE_{rel}^{CD} and $\Delta E_{rel}^{H_2O}$ for the CD and the water cluster taken separately. ΔE_{int}^{expl} is the interaction energy between the CD and its solvation sphere frozen at their optimized geometry.

	ΔE_{rel}^{tot}	ΔE_{rel}^{CD}	$\Delta E_{rel}^{H_2O}$	ΔE_{int}^{expl}
β-CD-C	0.00	0.00	0.00	-335.33
β-CD-O	7.39	19.89	30.00	-377.82

Experimental IR spectra of α-, β-, γ-cyclodextrins

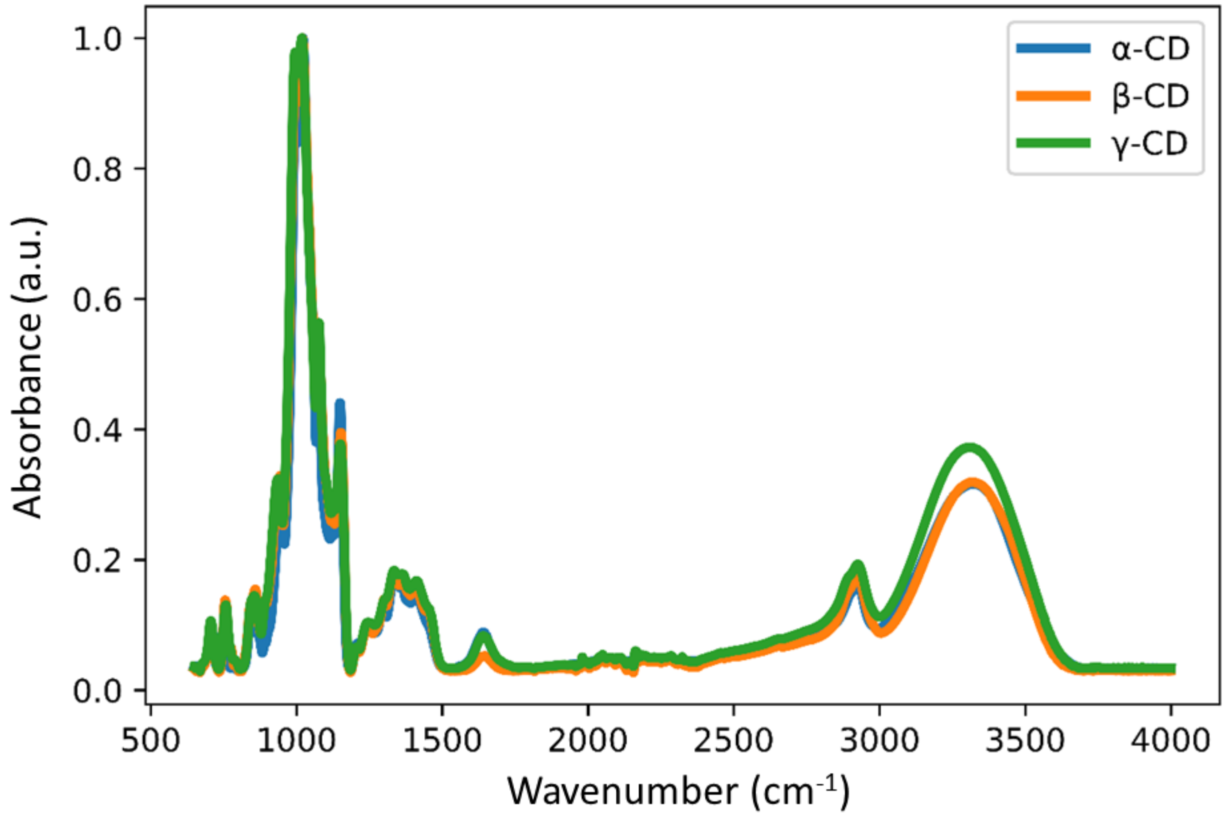

Figure S3: Superimposed experimental spectra of α-, β- and γ-cyclodextrin.
